# Consistent trait-temperature interactions drive butterfly phenology in both incidental and survey data

**DOI:** 10.1038/s41598-022-16104-7

**Published:** 2022-08-04

**Authors:** Elise A. Larsen, Michael W. Belitz, Robert P. Guralnick, Leslie Ries

**Affiliations:** 1 Department of Biology, Georgetown University, Regents Hall 501, Washington DC, 20057 USA; 2 Florida Museum of Natural History, University of Florida, Gainesville, FL 32611 USA; 3 University of Florida Biodiversity Institute, Gainesville, FL 32603 USA

**Keywords:** Phenology, Macroecology

## Abstract

Data availability limits phenological research at broad temporal and spatial extents. Butterflies are among the few taxa with broad-scale occurrence data, from both incidental reports and formal surveys. Incidental reports have biases that are challenging to address, but structured surveys are often limited seasonally and may not span full flight phenologies. Thus, how these data source compare in phenological analyses is unclear. We modeled butterfly phenology in relation to traits and climate using parallel analyses of incidental and survey data, to explore their shared utility and potential for analytical integration. One workflow aggregated “Pollard” surveys, where sites are visited multiple times per year; the other aggregated incidental data from online portals: iNaturalist and eButterfly. For 40 species, we estimated early (10%) and mid (50%) flight period metrics, and compared the spatiotemporal patterns and drivers of phenology across species and between datasets. For both datasets, inter-annual variability was best explained by temperature, and seasonal emergence was earlier for resident species overwintering at more advanced stages. Other traits related to habitat, feeding, dispersal, and voltinism had mixed or no impacts. Our results suggest that data integration can improve phenological research, and leveraging traits may predict phenology in poorly studied species.

## Introduction

Shifting phenology, the timing of life history events, is a primary response of organisms to changes in their environment, particularly related to climate^1,2^. Phenological patterns vary across space and time, often in ways that are predictable based on gradients in temperature and precipitation^3,4^ or static cues like photoperiod^5^. Yet not all species respond to environmental shifts in the same way^2^ and so when multiple species interact, differential shifts in phenology may cause mismatches in seasonal timing^6^. These mismatches may have demographic or even ecosystem consequences and phenological mismatches are currently a major focus of ecological and evolutionary research (e.g.,^4,7–9^).

Insect phenology has been shown to be particularly sensitive in terms of how organisms shift their timing to adjust to changing environments^3,10^. This is because insects are ectothermic and their developmental rate is thus driven largely by ambient temperature^10^. Because of their importance for agricultural systems, models that accumulate degrees within certain ranges (called growing degree day, or GDD, models) have been developed to predict local phenology of insects and have proven to be highly effective for both pest and non-pest species^11^. Butterflies are an excellent group for the study of insect phenology; their biology is well-known and they are primary consumers, the trophic level that has been found to be the most sensitive in terms of animal phenology^4^.

Studies of butterfly phenology range from detailed, mechanistic single-species studies (e.g.,^12–16^) to broader examinations of whole communities that assess consistency of responses across scales while also capturing species-specific variability in sensitivity (e.g.,^17–25^). Consistently and not surprisingly, these studies have found that many, but not all, butterfly species fly earlier in warmer years. Traditional timing of flight initiation (e.g., spring, summer, or fall flyers) has been found to be an important factor in phenological shifts^17–20,23^, with earlier flyers showing more sensitive shifts forward and later flyers sometimes shifting later. Overwinter stage was also shown to be important^17,19,20,23^ with the later developmental stages when overwintering (adult vs. pupa vs. larvae vs. egg) being associated with earlier and more sensitive flight period timing. Other traits that have been studied, such as hostplant breadth, mobility, and voltinism have had more mixed results^18,19,26,27^.

Phenological shifts are difficult to estimate because their detection is strongly influenced by the timing and structure of monitoring events. Thus, a critical component for all phenology studies is choosing an appropriate metric (or “yardstick”) and data set to detect change at different points along seasonal time-courses^6,28^. One metric of particular interest is onset, the first occurrence of adults each season. Yet onset is particularly difficult to estimate because it occurs, by definition, when population levels are at their smallest^28,29^. This challenge is compounded by monitoring data emerging from a variety of different observation protocols, each with particular biases that may obscure this hard-to-detect event. Also, onset timing may not be a useful metric for representing population phenology, as it may be driven by extreme conditions experienced by a small minority of individuals. One solution is to focus on an arbitrary threshold when a certain portion of earlier-flying individuals have been recorded (e.g., 10% or 25%). This approach provides a metric that is easily calculable, but will vary in ecological meaning, depending on the dynamics of a given population, particularly in relation to voltinism. Alternatively, mid-season metrics, (e.g. mean or median period) are often more robust to variation in data type and density^28^, but may be less meaningful in terms of ecological dynamics, such as mismatch.

Here, we focus on the Northeastern US butterfly community, comparing early (10%) adult flight and mid-season timing (50%) for data generated from two types of community (“citizen”) science inventories. These percentile metrics of phenology are based on data throughout the flight period, such that late season dynamics can result in changes to early and mid-season metrics. Thus, these metrics provide a different type of information on phenology than onset per se. While a percentile metric of the first generation would be the most direct comparison across species, many butterflies have overlapping generations and existing data do not lend themselves to discerning among generations. Thus, we consider percentile phenometrics across a species’ entire flight period. The interpretation of these metrics varies according to voltinism and abundance patterns across generations. For populations with significant population growth across a growing season, the relative abundance of later generations will shift the metrics toward the later generation, and be less representative of the initial flight. Still, early season metrics represent the emergence or arrival of the first 10% of adults within a region.

Our goal is to determine how these two popular and growing monitoring resources inform adult phenology at broad, regional scales as a means to understand their shared utility and potential for future, analytical integration. The first type of data emerges from networks of volunteers who carry out repeated surveys on established transects using academic-like protocols that were designed specifically to track broad patterns in butterfly abundance and timing^30^. These programs typically provide high quality data, including all observed target species, abundances, and metrics of effort; yet, such surveys are generally limited geographically because of the effort to initiate them, recruit volunteers, and retain them^31^. Of particular relevance to phenological studies, the timing of survey initiation each year will influence the ability to capture early-season dynamics^32^.

A second class of community science resource is incidental observations of butterflies posted to online platforms such as iNaturalist or eButterfly^33,34^. These platforms have few restrictions for inclusion and growth in participation has been phenomenal, leading to the highest spatial density of records compared to other monitoring programs, although their recent initiation means that the temporal scope of data is currently limited^35^. iNaturalist.org, for example, has nearly doubled the number of records collected every year since its inception in 2008. By 2014, participation had been slowly growing and, by that year, 1,738 community scientists added 19,598 butterfly observations globally. However, by 2020, 121,470 community scientists across the globe reported 838,080 butterfly observations, a greater than 40-fold increase in observations in just 6 years^36^. Almost all of these reports include a digital photograph voucher, and a sizable proportion have at least two agreed-upon identifications by other members of the iNaturalist community. iNaturalist considers these records as “research-grade”^37^.

This recent, explosive growth of incidental data provides significant potential for use in phenological analysis. However, accounting for variable effort across time and space for these resources is a substantial challenge^38–40^. Data without repeated site visits and where no information on effort or reports of absences (“presence-only” data) must account for recording bias, often by aggregating records at coarse grains (i.e. 10 km or higher) to achieve sufficient data density, though this also obscures local phenological variability^41^. Despite the challenges, with sufficient data density, deriving insights about phenology from presence-only data holds promise^28,42^. For example, Karlsson^17^ obtained high density of presence-only data from Sweden’s popular community science web portal (https://www.artportalen.se/), and found results consistent with other analyses of European butterflies (e.g.,^19,23,25^). The evidence suggests that with sufficient data density, both occurrence records and survey data can provide phenological data representative of a focal population.

Given potential biases in both data collection methods (structured surveys with inconsistent start dates or incidental reports that are presence-only), it is not clear how each data set differentially represents species’ phenology. Consistency of results between data sets is one benchmark that can be used to compare the validity of findings of multiple sources of data. Another benchmark to consider is whether findings conform to patterns reported elsewhere; results that diverge substantially from typical observations should be carefully rechecked for unaccounted data biases and the selection of appropriate modeling frameworks^40^. We present findings on phenological sensitivity from two sources of butterfly monitoring data. Given that developmental rates of Lepidoptera are directly driven by temperature, our specific a priori hypotheses are that flight phenology will advance where and when temperatures are warmer. Additionally, we predict that timing will be earlier and more sensitive for summer species that overwinter at more advanced stages (adult, pupae, larvae, then egg). We also explore the relationship between shifting phenology and other traits that have been found or suggested to potentially be important, including mobility, habitat association, hostplant breadth, and voltinism.

## Results

**Figure 1:**
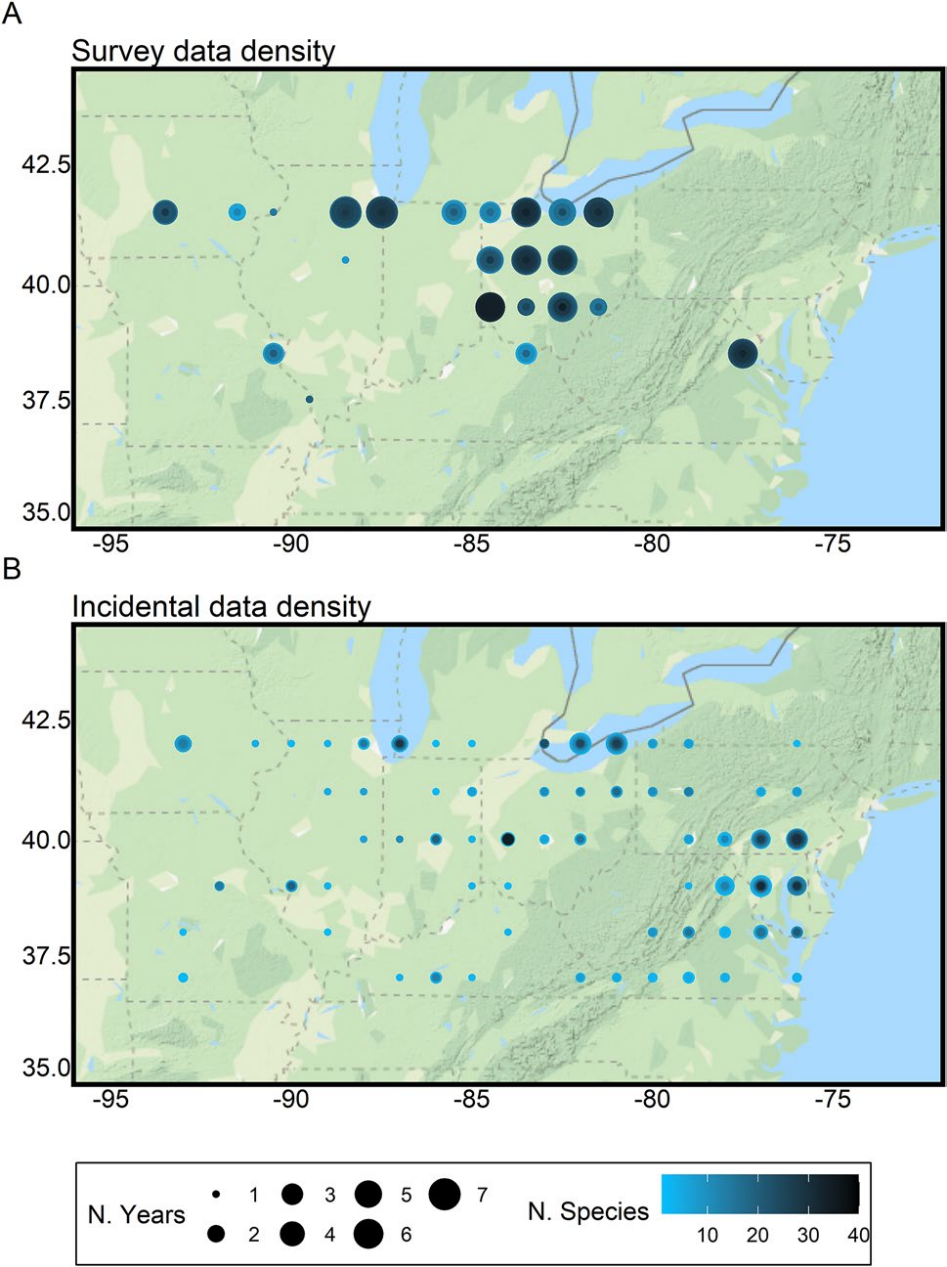
Study region with phenometric density from standardized surveys from the US butterfly monitoring network (**A**) and incidental data from iNaturalist and eButterfly (**B**). Phenometric density is described by concentric circles at one-degree grid cell centroids, where the diameter of the circle is number of years (1–7) and the color shade is the number of species with phenometrics for that number of years. The color of the inner-most ring of each circle represents the number of species with phenometrics in at least one year. Each successive ring outwards shows how many species had phenometrics in 2–7 years respectively. (**A**) demonstrates that butterfly survey phenometrics are currently limited geographically, but are taxonomically rich, while (**B**) shows that the incidental phenometrics are widespread but sufficient data were available for fewer species in many grid cells. This figure was made with the ggplot and ggmap packages in R^77^.

There were 114 possible grid cells in our study region (Fig. 1). For our 40 focal species during our 7-year study period (2012–2018), structured survey (“Pollard”) data were sufficient for phenological analysis of 1468 combinations of species, year, and grid cell, with an average of 14 grid cells per species (range 1–22). In each species, year, grid cell combination, flight periods were estimated from an average of 165 surveys (range 30–420) at 16 (range 2–41) sites, with abundances for the target species detected in 55 surveys (range 10–335). Incidental data were sufficient for phenological analysis of 1441 combinations of species, year, and grid cell, with an average of 17 grid cells per species (range 1–54) with at least 1 species in 69 of the possible 114 1-degree grid cells in the region (Fig. 1B). Flight periods in each grid cell-year combination were estimated from an average of 31 observations (range 10–347).

Further analysis was limited to 33 species and 15 grid cells in years 2014–2018 with comparable phenometrics. While species and grid cells included vary by year, metrics for a given species-cell-year were only included in analysis when metrics were available from both data sources. The vast majority of early flight period (10%) phenometrics occurred after the regionally identified “day 0” (Supplement 2 (S2) Fig. 1); “day 0” was defined as one week earlier than typical early flight period onset dates as per regional field guides (S1). Observed early flight dates often had a substantial lag compared to “day 0”, but this is not surprising since we were estimating a phenometric representing the early flight period as a comparison, as opposed to the earliest likely “onset”, against “day 0”. Among all species-cell-year combinations, only 1% and < 1% of estimated early season metrics occurred prior to “day 0” for survey and incidental data respectively. These could represent unusually early flight initiation, a rare colonization event from a warmer region, or even a misidentification. Overall, there was a large amount of variation in the lag from “day 0” to early flight period (10%) phenometrics, but without consistent bias in either dataset (S2 Fig. 1). Most estimates between incidental and survey data overlapped substantially and with the exception of only 5 out of 33 species (*Vanessa cardui*, *Pyrgus communis*, *Papilio troilus*, *Eurytides marcellus*, and *Atalopedes campestris*), incidental data did not have persistently earlier 10% day of year (DOY) estimates even though observational platforms have no constraints on the earliest submission dates. Note that structured surveys are not required to begin until June 1 (but are certainly allowed to start earlier) for most programs within the network of butterfly survey programs^31^.

**Figure 2:**
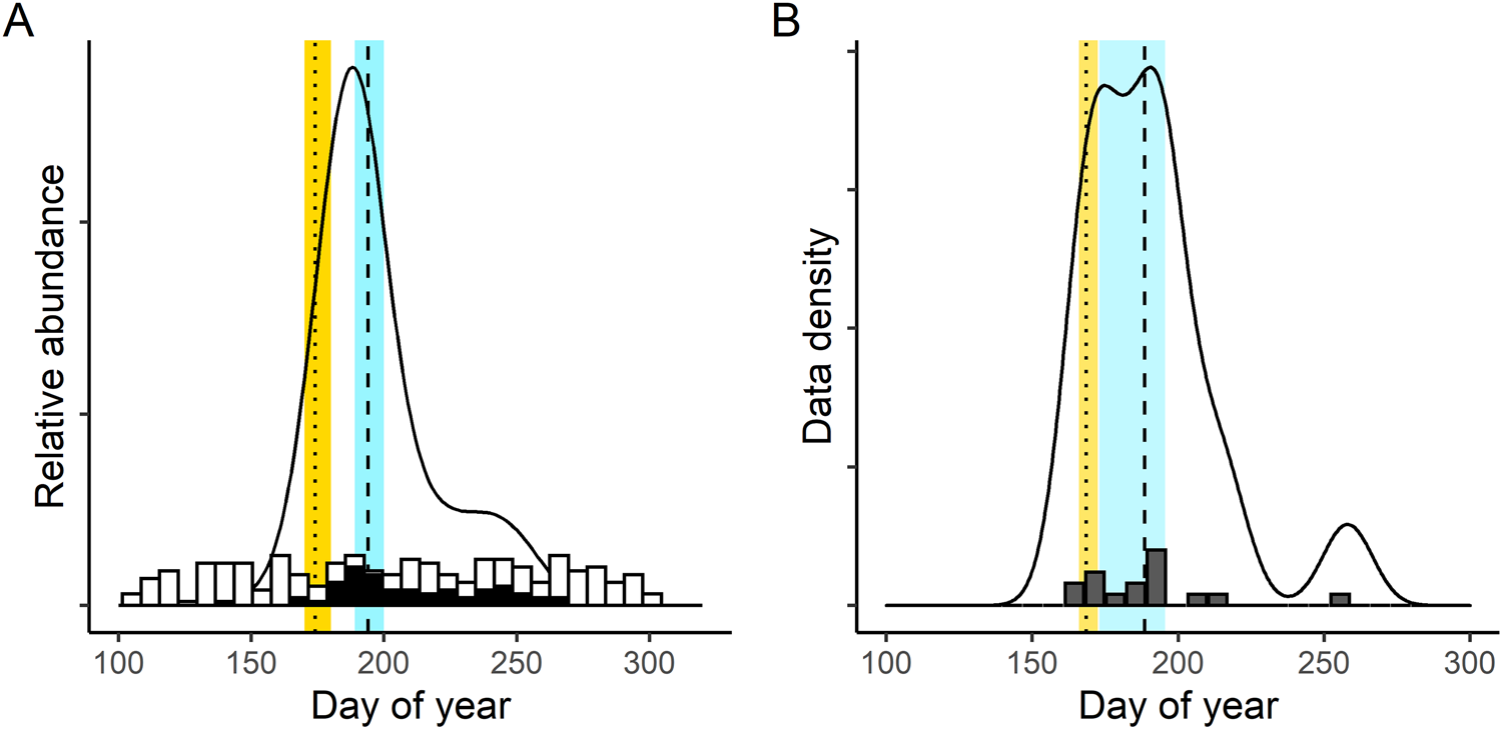
Survey (**A**) and incidental (**B**) data are compiled across day of year (DOY) for each species-year-grid cell unit to estimate a phenogram for the flight period, shown here for *Speyeria cybele*. In both panels, the black curve shows the flight phenograms calculated using appropriate analytical techniques: GAMs for survey data integrating phenological patterns across sites (**A**) and quantiles for incidental data aggregated across the grid cell (**B**). Our primary response variable for each analysis is the DOYs when 10% (dotted) and 50% (dashed) of butterflies have been counted, shown here with 95% confidence intervals [for 10% DOY estimate in yellow, 50% in blue]. The histogram in each panel shows underlying data: in (**A**) the number of surveys per week, where dark fill represents surveys which recorded the target species and in (**B**), the number of occurrence records per week.

Confidence intervals (CIs) for DOY estimates were often quite large, and this partially reflects the fact that phenology can vary substantially within a 1-degree grid cell. Within each cell, heterogeneity in landscape (elevation, slope, aspect, habitat, land use) and climate (temperature, precipitation) could lead to variation in flight period phenology. Also, CI size for estimates from survey data was inversely proportional to the number of surveys and directly proportional to number of sites for the survey dataset (Fig. 2, S2). For phenometrics using survey data, CIs were large with a mean of 45 (± 23) days, ranging from 2 to 155 for 10% DOY and a mean of 43 (± 21.5) days, ranging from 3 to 150 days for 50%. Using incidental data, CIs were smaller, averaging 36 (± 18) days (range 1–138 days) for early-season and 44 (+/− 22) days with a range of 0–168 for 50% DOY. CI size was not related to the number of observations for early season phenometrics but for mid-season, CI size was inversely proportional to number of observations (Fig. 2, S2). Higher species confusability correlated with higher CI size for three models (S2 Table 1).

**Figure 3:**
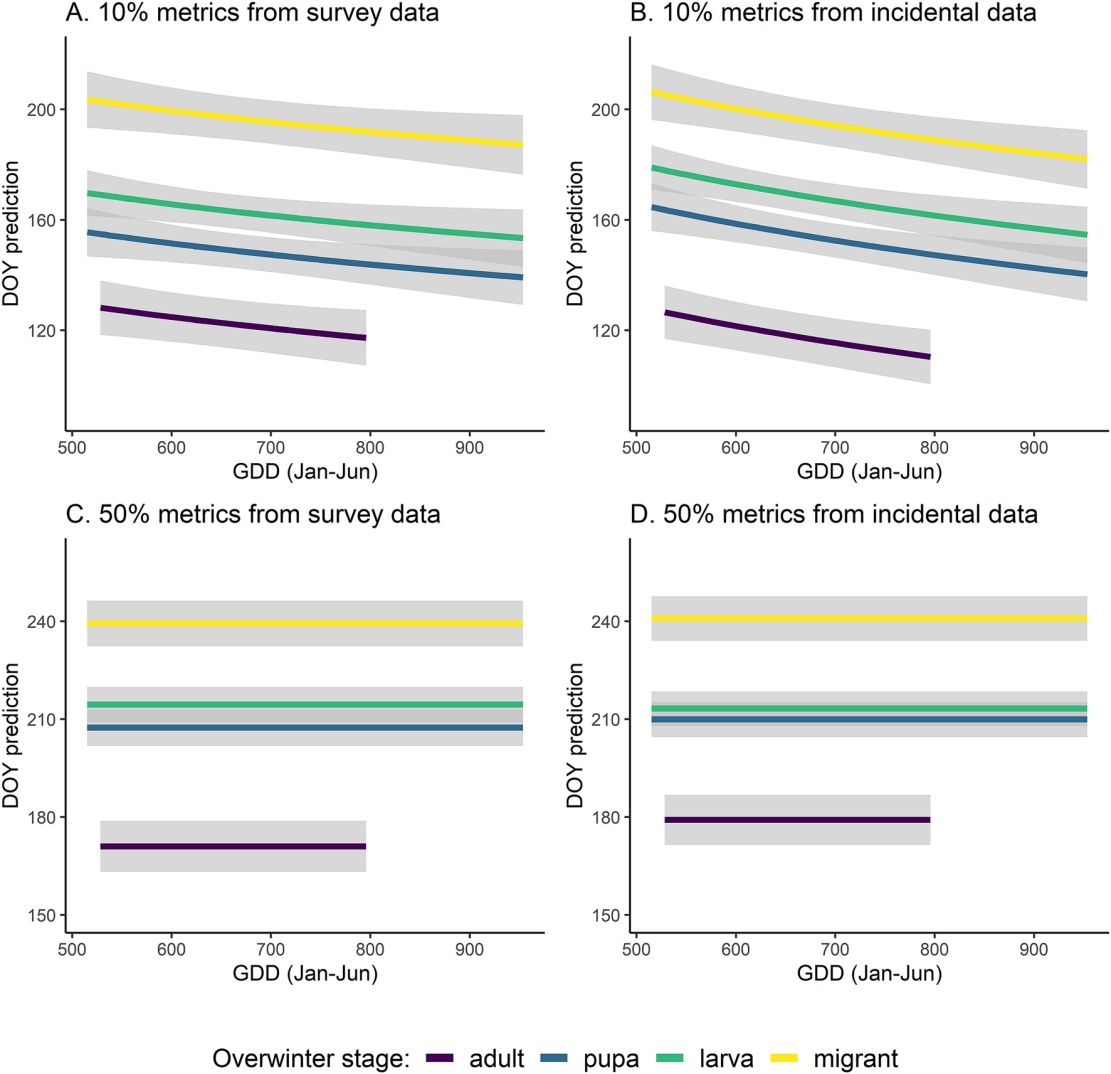
Model predictions for expected day-of-year (DOY) of early-season (10%) and mid-season (50%) phenology across species based on temperature (GDD) and overwinter strategy (by color: migrant species, and resident species overwintering as adults, pupae, larvae). Linear effect of log(GDD) shown on GDD axis. Model parameter estimates are in Table 1.

Mixed-model analyses of the influence of GDD and life-history traits were restricted to 32 species with complete trait data and 273 combinations of species flight periods for which phenometrics were estimable from both datasets. These flight periods fell within 15 grid cells and five years; while different grid cells were used for different species and in different years, each cell-year-species combination was only included in analysis if phenometrics were estimated from both survey and incidental data. Overall, early flight timing differed substantially by overwinter status (S2 Fig. 2). The best models for both the early (10%) and mid-season (50%) phenometrics included overwinter status for both survey and incidental data sets (Table 1). However, GDD was only retained for early season metrics (Fig. 3). There were no interactions between overwinter stage and GDD in any model. Host-plant specificity and local commonness were each included in a best-fit model but were not consistent between survey types. More common species showed earlier phenology metrics, but only in survey data; species with broader host plant breadth had later mid-season phenometrics, but only in incidental data (S2). These parameters were not as influential as GDD and overwinter stage. Other traits, including canopy associations, females laying eggs in clusters, voltinism, wing size, and mobility did not emerge as significant factors in any of the best-fit models.

**Table 1:** Parameter estimates from the best 10% emergence models.

	Best model using survey data			Best model using incidental data		
Parameter	Estimate	SE	p value	Estimate	SE	p value
log(GDD)	− 32.5	11.1	3.82 E−03	− 42.2	10.7	1.06 E−04
Adult diapause (0/1)	353.7	73.6	2.60 E−06	390.3	69.7	5.43 E−08
Pupal diapause (0/1)	382.1	74.3	5.34 E−07	427.6	70.5	4.63 E−09
Larval diapause (0/1)	398.8	73.8	1.46 E−07	442.2	70	1.14 E−09
Migrant (0/1)	431.9	74.6	2.01 E−08	469.8	70.8	1.88 E−10
Locally common (0/1)	− 23.8	9.4	1.48 E−02	NA	NA	NA
Marginal R^2^	0.43			0.42		
Conditional R^2^	0.5			0.53

Species identity also contributed to the variability explained by the model (Table 1, S2 Table 1). The best fit models included a random intercept by species identity, but no random species slope for GDD or random intercepts for detectability and confusability. Adding species identity added little explanatory power to early flight period models from either survey or incidental data. The model of early-season phenometrics (Table1) have marginal (fixed-effect only) R^2^ values of 0.43 for survey data and 0.42 for incidental data. Adding species identity increases the conditional (full-model) R^2^ only slightly, to 0.50 and 0.53 respectively. For mid-season analyses (S2); the marginal R^2^ is 0.40 for survey data and 0.37 for incidental data; adding species identity increases the conditional R^2^ more in these models, to 0.57 and 0.60 respectively.

## Discussion

Phenological patterns were remarkably similar between survey and incidental datasets, with both showing predicted patterns that align with what has generally been found for butterflies: emergence was earlier for species that overwinter at later developmental stages and when temperature is warmer (Fig. 3A,B). Because we could not a priori identify which of our datasets (survey or incidental) should be considered more reliable, our goal was to determine how closely the parameter estimates aligned with each other and with our a priori hypotheses. Fortunately, these two sources of data aligned with the expected patterns and each other. Our results present powerful confirmation for the ability of both types of data to provide robust information on phenological patterns, given sufficient data density and appropriate analytical frameworks. These results now provide a rationale for efforts to integrate both data types within a unified analysis (e.g.,^43^), which should be a goal of future methodological developments.

The reliability of phenological patterns generated from structured butterfly survey protocols are well vetted in other regions, particularly the UK^19,23^. Yet data from similar North American butterfly monitoring networks (BMNs) have not received the same level of attention. These programs generally start later and have fewer visits per season, so it was not clear whether shifts in early-season patterns would be detected in our study region. To date, only data from the Ohio Butterfly Monitoring Network have been used due to its inclusion of earlier years and its frequent visits^31^; previous results were generally consistent with earlier flight initiation during warmer years^21^, although delays were noted when warmer years interacted with urban heat islands^20^.

Unlike survey datasets, the ability of incidental data to inform early season phenology is much less tested and more controversial because of the challenge of accounting for biases when effort is unknown^28,40^. When appropriate filtering and presence-only methods are used to generate phenometrics from incidental data, results have often been consistent with expectations, including from Sweden^17^, North America^18^, and France^42^. Other studies using incidental data have failed to find consistent patterns when compared to surveys^44,45^, but these relied on museum specimens, which are generally sparser and frequently more biased with regard to flight period phenology than community-science generated incidental records^46^. Inconsistent or counter-intuitive results may also have resulted from poorly vetted data or inappropriate analyses^40^. Ultimately, assessing the utility of incidental data in insect phenology has been difficult because of the lack of best practices for addressing biases and using the most appropriate metrics (but see^28,42^). Analysis of percentile metrics require a different perspective for interpretation than an “onset” metric, as well. While interpretation of a percentile metric is straightforward for univoltine species, it can be more difficult for bivoltine and multivoltine species, particularly those whose abundances vary across generations, as is common in butterflies. For example, a 50% metric for a bivoltine population may occur on a date between generations, when no butterflies are flying. Because percentile metrics are affected by the dynamics across the entire season, changes to early season metrics could be due to varying abundances late in the flight period, or flight period length, rather than changes in the early flight period.

Beyond the methodological comparison, our results provide strong evidence supporting the key role of temperature and life history traits as controls on butterfly phenology. Overwinter strategy combined with GDD was sufficient to capture much of the variability surrounding early-season (10%) timing (Fig. 3). Seasonal emergence is earlier for resident species that overwinter at more advanced stages, which aligns with developmental time requirements following winter diapause. Also, species that overwinter in other areas and migrate to the region tend to arrive after resident species have initiated flights. Surprisingly, species identity and other traits such as mobility added very little predictive power, which suggests that for holometabolous insects, spring phenology may be predictable based on information that is often known: overwinter stage of the target species and local temperature profiles. Thus, even for species whose biology is not as well known, such as non-butterfly moths, flies, and beetles, emergence and arrival patterns may be predictable if overwintering stage is known. Although natural history information is often lacking for these groups, this still opens the door for substantial expansion of phenological studies across different taxa, an important goal in an age of insect declines^47^. These results amplify recent work that examined phenology across multiple insect orders (Coleoptera, Diptera, Hemiptera, Hymenoptera, Lepidoptera, and Odonata) which also found that overwinter stage was a strong and important predictor for initiation, termination, and duration of adult insect activity^48^.

We included the confusability and commonness covariates in our models to examine if these species traits would differentially bias phenometrics based on survey and incidental data. Only in the survey dataset did common species have advanced early-season phenometrics. Common species may have advanced phenometrics because observers are more likely to catch the beginning of phenological events in species with higher relative abundance. Survey data may accentuate this bias because the collection method is focused on gathering abundance data and due to pervasive false negatives in monitoring data differentially affecting less common species^49^. Conversely, recorders of incidental data may put more effort into adding new species to their yearly observation list. While survey locations often target a variety of specific habitats used by butterflies, incidental data records are disproportionately in places with greater human activity; these approaches may each bias taxonomic coverage in different ways. Continued examination of the influences of imperfect detection, relative abundance, and their potential interactions on estimating phenology using community science data is warranted.

While we were able to derive clear drivers of phenological variability, estimates from both datasets varied widely and confidence intervals were often large. Increasing effort measured either via number of surveys or observers is critical for determining phenology with precision. Researchers need to thoughtfully consider data density thresholds needed to determine phenology at a precision appropriate for their questions. To develop robust estimates of phenology, data density must be sufficient across the environmental gradients of interest^40^. Spatial aggregation is common and frequently necessary in macroecological studies; however, the relationship between number of survey sites and size of confidence intervals in survey data phenometrics makes clear that such aggregation can hide important local scale variation. Spatial scale can influence early and late season phenometrics^50^, and aggregation must be considered with respect to the ecological questions and the grain of environmental drivers being considered. Given the coarse spatial aggregation of this study (~ 110 km × 85 km cells), it is not surprising that the confidence intervals on phenometrics are frequently large.

This study highlights both the value and important limitations of these datasets. The lack of restrictions in incidental data collection can lead to broad spatial and taxonomic coverage and with sufficient data density, can capture important signals in phenology for many species. Survey methods have more limited spatial coverage but provide a structure and consistency that is itself valuable, tracking individual populations across time^51,52^. Additionally, survey data currently have greater temporal depth since widespread collection of incidental data is a relatively new activity that has expanded with mobile app development. Each dataset contains biases related to taxonomy, location, and weather, which may impact their value for certain research questions according to how these biases relate to the factors or gradients of interest.

The similarity of the spatiotemporal patterns in phenology generated by these two data sources suggests there is potential in integrating across data types to model phenology. Data integration techniques can expand not only the scale and scope of analyses^53^, but can also improve the accuracy and precision of estimates^43,54^. Methods that integrate multiple data sources into single modeling frameworks have increased over recent years, particularly for species distribution modeling^55^ and population models^43^. Specific methods to integrate phenological data are underdeveloped but the data are increasingly available. Development of phenology-centered data integration approaches will expand our ability to understand phenological patterns, as well as the drivers and potential consequences of these patterns.

Accurate and precise phenological metrics of butterflies are of urgent concern given reports of broad butterfly declines^56–58^ and recent results suggesting phenological shifts are associated with overall abundance trends^26,59^. Warmer temperatures are also leading to additional generations in certain multivoltine butterfly species, which may lead to unexpected shifts in median phenology, as well as affecting demographic outcomes^60^. While the focus here has been on early season and median timing across broad spatial and taxonomic scales, enhanced ability to capture number of broods over a season using incidental reporting or, better yet, integrated with structured survey data, may be possible but require new analytical approaches. Such new methods that leverage combined data sources hold enormous promise in expanding our capacity to not only understand drivers of spatial and temporal changes in phenology, but also better predict divergent future dynamics in the face of accelerating global change.

## Materials and methods

We examined phenological patterns in a region of the northeastern US bounded by 36 N and 42 N latitude, and 94 W and 76 W longitude (Fig. 1). Comparisons were between two community science datasets: (1) survey data from structured monitoring networks whose members conduct regular, repeated visits to monitoring sites using similar (“Pollard”) protocols, and (2) incidental data from iNaturalist and eButterfly. Forty butterfly species were selected by data density thresholds (described below) in one or both datasets. Species were identified by overwinter strategy, as either migrant or resident. Residents were further identified by overwinter life stage and information about other traits were compiled from published sources^61–69^ and used as covariates in our analyses (see Supplement 1 (S1) for focal trait descriptions, data by species, and sources). We include observations over the years 2012–2018, spatially aggregating data using 1-degree grid cells, which correspond to 111 km latitude, and 83–90 km longitude (Fig. 1).

Annual phenological metrics were estimated for early (10%) and mid (50%) flight periods for each dataset separately (Fig. 2). Note that data density thresholds meant that most species retained for analysis were summer rather than predominantly spring or fall flyers (S1). Structured surveys came from the following butterfly monitoring networks (BMNs): Illinois, Ohio, Iowa, Michigan, Missouri, Occoquan Bay (Northern VA). Survey seasons are not required to start until June for most programs but may start earlier^31^. The Ohio program more typically begins surveys in May and the Occoquan program operates year-round (they also survey birds). While each program has the ability to customize methods, training, and survey intensity, the similar data structures allow for integration of data across programs to produce a unified, regional analysis (following^32^). Survey data were extracted for grid cell and year combinations with at least 10 surveys completed each year across 3 or more sites and the number of species analyzable in each grid is shown in Fig. 1A.

Species-specific butterfly flight periods were estimated with regional generalized additive models (GAMs) within grid cells using the rbms package in R^32,70^. This GAM approach assumes that a species’ phenology is synchronized within a grid cell, but allows local abundance to vary across sites. Integrating the area under the phenology curve estimates relative abundance across time in “butterfly days”^32^. For each species, year, and grid cell in this study, we compiled data across sites and extracted the day of year (DOY) on which the area under the GAM curve reached 10% and 50% of the total area (Fig. 2A). We calculated 95% confidence intervals for each DOY phenological estimate (colored bands in Fig. 2A) by bootstrapping, resampling individual surveys (unique site and date) using the GAM model and the boot package in R. To avoid biased metrics for species present prior to local start dates for structured surveys, we estimated phenometrics for species present in at least four surveys across all sites, and not detected in the first survey of the year for at least one site at which the focal species was subsequently observed in the given year.

Incidental occurrence data were provided by community science volunteers submitting to iNaturalist and eButterfly. For iNaturalist records, we downloaded research grade observations^71^. To be considered research grade, observations must be georeferenced, include photos, have a date, not be recorded as cultivated, and at least two users must agree on taxon identity^36^. While species identifications are always prone to some level of error, many experts are active on the platform, and the quality of identifications is often high, especially for the common North American species examined here. As a check of data accuracy, we examined 9,974 images labeled as *Danaus plexippus* (monarch butterfly) that were scored as part of this project for identification accuracy and found that only two images were incorrectly identified. These images were for *Limenitis archippus* (the viceroy butterfly), a mimic of monarchs with a very similar appearance. For all research grade iNaturalist butterfly records in our species list, we also reviewed each image linked to the iNaturalist dataset to confirm that only adults were used in our analysis. We then combined iNaturalist incidental records with those from eButterfly. eButterfly is a community science platform that allows users to upload either single observations of butterflies or counts of every butterfly species seen during an observation outing (Prudic et al. 2017). eButterfly also encourages users to list the number of observers, time spent observing, and distance traveled, which can be used to quantify survey effort. However, most submissions do not include these, so we treated all eButterfly data as incidental. eButterfly performs quality control on observations by verifying that observations occur within their known ranges.

A final, combined dataset of incidental records from iNaturalist and eButterfly was produced and data density calculated for each grid cell (Fig. 1B). Observations across platforms were deemed duplicates if records were of the same species and had the same date, longitude, and latitude values, and only one record was retained. Phenometrics were calculated for incidental data using quantiles (Fig. 2B), where abundances divided across flight periods are put into equal bins. Given the likelihood of long flight periods with multiple generations for some of our species, quantiles are the least biased method for estimating both early and mid-flight period phenology^28^. Phenometrics were estimated for species, year, and grid cell combinations with at least 10 occurrence records, which based on simulations provide usable estimates^28^. We estimated the day of year (DOY) of the 10% and 50% quantiles for the combined incidental dataset. We calculated 95% confidence intervals for each DOY phenological estimate by bootstrapping the quantile estimates with different combinations of occurrence records (colored bands in Fig. 2B).

We identified species-cell-year combinations where phenometrics were estimable from both survey and incidental datasets. By filtering phenometrics to this subset, we were able to submit derived phenometrics from both survey and incidental datasets as response variables in identical GLMM analyses; we then compared the resultant patterns in relation to species traits and climate. However, we first produced a common-sense analysis to check for consistency among each dataset and known phenology from field guides. We calculated the differences between the early season (10%) metric and a timepoint we designated as “day 0” for each species-grid cell combination. Specifically, “day 0” was estimated to be one week prior to typical flight initiation times for each species in each region, as estimated from regional field guides (S1;^61–69^). We also tested for systematic bias, examined overlaps of 95% confidence intervals, and determined whether the size of those intervals is affected by sampling intensity metrics as well as species traits.

We examined spatio-temporal patterns in phenology for each dataset using a mixed effects linear model with 10% and 50% DOY as the response variables (Fig. 2). For both models, we used accumulated GDD and life history traits (Table 1) as explanatory, fixed variables and factors related to observation (detectability and confusability) and species identity as explanatory random variables. We accumulated GDD from January 1 to June 30 for each cell-year combination to provide an index of the relative amount of energy available for growth spatially across grid cells and temporally across years. Daily mean temperature values were extracted at 1 km resolution from the Daymet climate data product^72^ and averaged across focal one-degree grid cells for analysis. GDD were calculated from those mean values as a single-sine approximation accumulating degrees within the commonly-used generic thresholds of 10 °C and 30 °C as base temperature and maximum temperature, respectively^73^. All analyses were conducted using R version 4.0.2^74^. Model selection was conducted using AIC in package lmerTest^75^, and pseudo-R2 values (both marginal and conditional) were estimated using the MuMIn package, following^76^.

## Supplementary Information

Supplementary Information 1.

Supplementary Information 2.

## Data Availability

Data sets and R code utilized for this research are as follows: (1) Daymet data: https://doi.org/10.3334/ORNLDAAC/1328. (2) GBIF Occurrence download: https://doi.org/10.15468/dl.1erh15. (3) Life history data: in Supplemental files and github repo. (4) Combined occurrence & BMN survey phenometrics dataset: github repository release archived at 10.5281/zenodo.6946142. (5) All R code files are also available at 10.5281/zenodo.6946142. There is minimal novel code.

## References

[1] Parmesan C (2006). Ecological and evolutionary responses to recent climate change. Annu. Rev. Ecol. Syst..

[2] Forrest J, Miller-Rushing AJ (2010). Toward a synthetic understanding of the role of phenology in ecology and evolution. Philos. Trans. R. Soc. B Biol. Sci..

[3] Cohen, J. M., Lajeunesse, M. J. & Rohr, J. R. A global synthesis of animal phenological responses to climate change/631/158/2165/2457/631/158/2039/129/141/139 letter. *Nat. Clim. Chang.* **8** (2018).

[4] Thackeray SJ (2016). Phenological sensitivity to climate across taxa and trophic levels. Nature.

[5] Mushegian AA (2021). Ecological mechanism of climate-mediated selection in a rapidly evolving invasive species. Ecol. Lett..

[6] Visser ME, Both C (2005). Shifts in phenology due to global climate change: the need for a yardstick. Proc. R. Soc. B Biol. Sci..

[7] Mayor SJ (2017). Increasing phenological asynchrony between spring green-up and arrival of migratory birds. Sci. Rep..

[8] Beard, K. H., Kelsey, K. C., Leffler, A. J. & Welker, J. M. The missing angle: Ecosystem consequences of phenological mismatch. *Trends Ecol. Evol*. **34** (2019). 10.1016/j.tree.2019.07.019 31451305

[9] Youngflesh C (2021). Migratory strategy drives species-level variation in bird sensitivity to vegetation green-up. Nat. Ecol. Evol..

[10] Forrest, J. R. Complex responses of insect phenology to climate change. *Curr. Opin. Insect Sci*. **17** (2016). 10.1016/j.cois.2016.07.002 27720073

[11] Crimmins, T. M. *et al.* Short-term forecasts of insect phenology inform pest management. *Ann. Entomol. Soc. Am.* **113** (2020).

[12] Brakefield, P. M. Geographical variability in, and temperature effects on, the phenology of Maniola jurtina and Pyronia tithonus (Lepidoptera, Satyrinae) in England and Wales. *Ecol. Entomol.* **12** (1987).

[13] Dell D, Sparks TH, Dennis RLH (2005). Climate change and the effect of increasing spring temperatures on emergence dates of the butterfly *Apatura iris* (Lepidoptera: Nymphalidae). Eur. J. Entomol..

[14] Van Der Kolk, H. J., Wallisdevries, M. F. & Van Vliet, A. J. H. Using a phenological network to assess weather influences on first appearance of butterflies in the Netherlands. *Ecol. Indic.* **69** (2016).

[15] Abarca, M. *et al.* Inclusion of host quality data improves predictions of herbivore phenology. *Entomol. Exp. Appl.* **166** (2018).

[16] Abarca, M. & Lill, J. T. Latitudinal variation in the phenological responses of eastern tent caterpillars and their egg parasitoids. *Ecol. Entomol.* **44** (2019).

[17] Karlsson B (2014). Extended season for northern butterflies. Int. J. Biometeorol..

[18] Kharouba, H. M., Paquette, S. R., Kerr, J. T. & Vellend, M. Predicting the sensitivity of butterfly phenology to temperature over the past century. *Glob. Chang. Biol.* **20** (2014). 10.1111/gcb.12429 24249425

[19] Diamond, S. E., Frame, A. M., Martin, R. A. & Buckley, L. B. Species’ traits predict phenological responses to climate change in butterflies. *Ecology* **92** (2011). 10.1890/10-1594.1 21661561

[20] Diamond, S. E. *et al.* Unexpected phenological responses of butterflies to the interaction of urbanization and geographic temperature. *Ecology* **95** (2014).

[21] Cayton HL, Haddad NM, Gross K, Diamond SE, Ries L (2015). Do growing degree days predict phenology across butterfly species?. Ecology.

[22] Stewart, J. E., Illán, J. G., Richards, S. A., Gutiérrez, D. & Wilson, R. J. Linking inter-annual variation in environment, phenology, and abundance for a montane butterfly community. *Ecology* **101** (2020). 10.1002/ecy.2906 PMC9285533 31560801

[23] Roy, D. B. *et al.* Similarities in butterfly emergence dates among populations suggest local adaptation to climate. *Glob. Chang. Biol.* **21** (2015). 10.1111/gcb.12920 PMC4744750 26390228

[24] Dennis RLH (2010). Turnover and trends in butterfly communities on two British tidal islands: Stochastic influences and deterministic factors. J. Biogeogr..

[25] Sparks, T. H. & Yates, T. J. The effect of spring temperature on the appearance dates of British butterflies 1883–1993. *Ecography (Cop.).* **20** (1997).

[26] Michielini JP, Dopman EB, Crone EE (2021). Changes in flight period predict trends in abundance of Massachusetts butterflies. Ecol. Lett..

[27] Zografou, K. *et al.* Species traits affect phenological responses to climate change in a butterfly community. *Sci. Rep.* **11** (2021). 10.1038/s41598-021-82723-1 PMC7870830 33558563

[28] Belitz MW, Larsen EA, Ries L, Guralnick RP (2020). The accuracy of phenology estimators for use with sparsely sampled presence-only observations. Methods Ecol. Evol..

[29] Van Strien AJ, Plantenga WF, Soldaat LL, Van Swaay CAM, WallisDeVries MF (2008). Bias in phenology assessments based on first appearance data of butterflies. Oecologia.

[30] Pollard, E. A method for assessing changes in the abundance of butterflies. *Biol. Conserv.* **12** (1977).

[31] Taron, D. & Ries, L. Butterfly Monitoring for Conservation. in *Butterfly Conservation in North America* 35–57 (Springer Netherlands, 2015). 10.1007/978-94-017-9852-5_3.

[32] Schmucki R (2016). A regionally informed abundance index for supporting integrative analyses across butterfly monitoring schemes. J. Appl. Ecol..

[33] Prudic K, Oliver J, Brown B, Long E (2018). Comparisons of citizen science data-gathering approaches to evaluate urban butterfly diversity. Insects.

[34] Prudic, K. L. *et al.* eButterfly: Leveraging massive online citizen science for butterfly conservation. *Insects* **8** (2017). 10.3390/insects8020053 PMC5492067 28524117

[35] Barve, V. V. *et al.* Methods for broad-scale plant phenology assessments using citizen scientists’ photographs. *Appl. Plant Sci.* **8** (2020). 10.1002/aps3.11315 PMC6976896 31993257

[36] Seltzer, C. Making biodiversity data social, shareable, and scalable: Reflections on iNaturalist & citizen science. *Biodivers. Inf. Sci. Stand.* **3** (2019).

[37] Wittmann, J., Girman, D. & Crocker, D. Using inaturalist in a coverboard protocol to measure data quality: Suggestions for project design. *Citiz. Sci. Theory Pract.* **4** (2019).

[38] Dorazio, R. M. Accounting for imperfect detection and survey bias in statistical analysis of presence-only data. *Glob. Ecol. Biogeogr.* **23** (2014).

[39] Ries, L., Zipkin, E. F. & Guralnick, R. P. Tracking trends in monarch abundance over the 20th century is currently impossible using museum records. In *Proceedings of the National Academy of Sciences of the United States of America* vol. 116 (2019). 10.1073/pnas.1904807116 PMC6628677 31291704

[40] Larsen EA, Shirey V (2021). Method matters: Pitfalls in analysing phenology from occurrence records. Ecol. Lett..

[41] de Keyzer, C. W., Rafferty, N. E., Inouye, D. W. & Thomson, J. D. Confounding effects of spatial variation on shifts in phenology. *Glob. Chang. Biol.* **23** (2017). 10.1111/gcb.13472 27550575

[42] Cima V (2020). A test of six simple indices to display the phenology of butterflies using a large multi-source database. Ecol. Indic..

[43] Zipkin, E. F. *et al.* Addressing data integration challenges to link ecological processes across scales. *Front. Ecol. Environ.* **19** (2021).

[44] Polgar, C. A., Primack, R. B., Williams, E. H., Stichter, S. & Hitchcock, C. Climate effects on the flight period of Lycaenid butterflies in Massachusetts. *Biol. Conserv.* **160** (2013).

[45] Brooks SJ (2017). The influence of life history traits on the phenological response of British butterflies to climate variability since the late-19th century. Ecography (Cop.).

[46] van Strien, A. J., van Swaay, C. A. M., van Strien-van Liempt, W. T. F. H., Poot, M. J. M. & WallisDeVries, M. F. Over a century of data reveal more than 80% decline in butterflies in the Netherlands. *Biol. Conserv.* **234** (2019).

[47] Boggs, C. L. The fingerprints of global climate change on insect populations. *Curr. Opin. Insect Sci.* **17** (2016). 10.1016/j.cois.2016.07.004 27720076

[48] Belitz, M. *et al.* Climate drivers of adult insect activity are conditioned by life history traits. *Authorea Prepr.* (2021). 10.1111/ele.13889 34636143

[49] Kellner, K. F. & Swihart, R. K. Accounting for imperfect detection in ecology: A quantitative review. *PLoS ONE* **9** (2014). 10.1371/journal.pone.0111436 PMC4214722 25356904

[50] Park, D. S., Newman, E. A. & Breckheimer, I. K. Scale gaps in landscape phenology: challenges and opportunities. *Trends Ecol. Evol.* **36** (2021). 10.1016/j.tree.2021.04.008 33972119

[51] Kerr JT, Vincent R, Currie DJ (1998). Lepidopteran richness patterns in North America. Écoscience.

[52] Taylor, S. D., Meiners, J. M., Riemer, K., Orr, M. C. & White, E. P. Comparison of large-scale citizen science data and long-term study data for phenology modeling. *Ecology* **100** (2019). 10.1002/ecy.2568 PMC7378950 30499218

[53] Isaac, N. J. B. *et al.* Data integration for large-scale models of species distributions. *Trends Ecol. Evol.* **35** (2020). 10.1016/j.tree.2019.08.006 31676190

[54] Miller, D. A. W., Pacifici, K., Sanderlin, J. S. & Reich, B. J. The recent past and promising future for data integration methods to estimate species’ distributions. *Methods Ecol. Evol.* **10** (2019).

[55] Fletcher RJ (2019). A practical guide for combining data to model species distributions. Ecology.

[56] Wepprich T, Adrion JR, Ries L, Wiedmann J, Haddad NM (2019). Butterfly abundance declines over 20 years of systematic monitoring in Ohio, USA. bioRxiv.

[57] Crossley MS (2021). Recent climate change is creating hotspots of butterfly increase and decline across North America. Glob. Chang. Biol..

[58] Forister ML (2021). Fewer butterflies seen by community scientists across the warming and drying landscapes of the American West. Science (80-).

[59] Macgregor, C. J. *et al.* Climate-induced phenology shifts linked to range expansions in species with multiple reproductive cycles per year. *Nat. Commun.* **10**, (2019). 10.1038/s41467-019-12479-w PMC6813360 31649267

[60] Kerr, N. Z. *et al.* Developmental trap or demographic bonanza? Opposing consequences of earlier phenology in a changing climate for a multivoltine butterfly. *Glob. Chang. Biol.* **26**, (2020). 10.1111/gcb.14959 31833162

[61] Belth, J. E. *Butterflies of Indiana: A field guide*. *Butterflies of Indiana: A Field Guide* (2012).

[62] Betros B (2008). A Photographic Field Guide to the Butterflies in the Kansas City Region.

[63] Bouseman, J. K., Sternburg, J. G. & Wiker, J. R. *Field guide to the skipper butterflies of Illinois*. (Illinois Natural History Survey Manual 11, 2006).

[64] Clark, A. H. The butterflies of the District of Columbia and vicinity. *Bull. United States Natl. Museum* (1932).

[65] Glassberg J (1993). Butterflies through Binoculars: Boston—New York—Washington Region.

[66] Glassberg J (1999). Butterflies through Binoculars: The East—A Field Guide to the Butterflies of Eastern North America.

[67] Iftner DC, Shuey JA, Calhoun JV (1992). Butterflies and skippers of Ohio.

[68] Jeffords MR, Post SL, Wiker J (2019). Butterflies of Illinois: a field guide.

[69] Schlicht DW, Downey JC, Nekola JC (2007). The butterflies of Iowa.

[70] Schmucki, R., Harrower, C. A. & Dennis, E. B. rbms: Computing generalised abundance indices for butterfly monitoring count data. R package version 1.1.0. https://github.com/RetoSchmucki/rbms (2021).

[71] GBIF. *GBIF Occurrence download.*10.15468/dl.1erh15 (2019).

[72] Thornton, P. E. *et al.* Daymet: Daily surface weather data on a 1-km grid for North America, version 3. *ORNL DAAC*. (Oak Ridge, TN, 2017).

[73] Baskerville, G. L. & Emin, P. Rapid estimation of heat accumulation from maximum and minimum temperatures. *Ecology* **50**, (1969).

[74] R Development Core Team, R. R: A Language and Environment for Statistical Computing. *R Foundation for Statistical Computing* vol. 1 409 (2011).

[75] Kuznetsova, A., Brockhoff, P. B. & Christensen, R. H. B. lmerTest: Tests for random and fixed effects for linear mixed effect models (lmer objects of lme4 package). *R package version* (2014).

[76] Nakagawa, S. & Schielzeth, H. A general and simple method for obtaining R2 from generalized linear mixed-effects models. *Methods Ecol. Evol.* **4** (2013).

[77] Kahle, D. & Wickham, H. ggmap: Spatial visualization with ggplot2. *R J* **5** (2013).

