# Supplementary material for: Consistent trait-temperature interactions drive butterfly phenology in both incidental and survey data
Source: Sci Rep. 2022 Aug 4;12:13370. doi: 10.1038/s41598-022-16104-7 (PMC9352721; doi:10.1038/s41598-022-16104-7)
Supplement: Supplementary file 1 — Supplementary Information 1. [file 41598_2022_16104_MOESM1_ESM.pdf]

Consistent trait-temperature interactions drive butterfly phenology in both incidental and survey data

Supplement 1. Species trait data

Metadata and data table for species traits are available in the supplemental excel table and the code repository. Species traits were collated from published field guides [62-70].

Table legends:

Supplement 1 Table 1: This table provides the metadata (data types and descriptions) for species trait fields collated in Supplement 1 Table 2 and used in this analysis. Data types are text, boolean, integer, or [non-integer] numeric. Descriptions provide information for interpreting trait fields, including measurement units and explanations of numeric scales.

Supplement 1 Table 2: This table provides the species-specific life history traits used for this analysis. Field descriptions and units are provided in Supplement 1 Table 1.

Supplement 2. Supplemental analysis and results

Confidence interval analysis

We examined how confidence interval (CI) size varies with both species traits and sampling intensity to better understand how these factors relate to phenometric precision. For incidental data, we tested whether precision varies with the number of species observations available for metric estimation. For survey data, we tested whether precision varies with the

number of sites surveyed, the total number of surveys, or the number of species observations. For all metrics, we included wing size (mean or maximum), detectability, and confusability. Because of strong correlations among mean wing size, maximum wing size, and detectability, we tested each of these in separate models. All statistical models included a random intercept for species and were conducted using lmer and lmerTest packages in R version 4.0.2.

Species traits did influence confidence interval size. Confusability was a significant predictor of CI size for both 10% and 50% phenometrics from survey data, but only 10% phenometrics from incidental data (S2 Table 1). As expected, in all of these models, higher confusability was correlated with larger confidence intervals. Wing size (maximum) was inversely proportional to CI size for 10% metrics, but only in survey data. It’s unclear why larger butterflies would have less precise emergence metrics, though perhaps it relates to the geographic scope of survey sites and the home ranges of butterflies of different sizes.

Sampling intensity was consistently a significant predictor of phenometric precision from survey data; CI size was directly correlated to number of sites and inversely proportional to number of surveys. For phenometrics from incidental data, sample size was only retained in the model for CI size in the mid-season phenometrics (S2 Table 1). Interestingly, much more variance in CI size is explained for survey data metrics (30% and 20% for emergence and mid-season metrics respectively) than for incidental data metrics (4% and 8%). The random intercept for species also explained a small portion of the variance in CI sizes in most models.

<u>Additional results from phenometric analysis with traits and GDD</u>

The main text describes in detail the analysis of phenological estimates for emergence (10%) and mid-season (50%) phenology. Here we present additional results from this analysis.

S2 Table 2 provides a summary of the best-fit models for 50% phenological metrics. S2 Fig. 1 shows the lags between “day 0” and emergence metrics by species. S2 Fig. 2 shows the emergence and mid-season phenometrics grouped by overwinter stage.

Supplement 2 Tables and Figures.

S2 Table 1. Results of mixed effects models for confidence interval size.

	**Best 10% CI model**			**Best 50% CI model**		
Parameter	Estimate	SE	p value	Estimate	SE	p value
**Incidental data**						
Confusability	6.062	2.50	2.44 E-02	NA	NA	NA
# observations	NA	NA	NA	-0.26	0.05	6.65 E-07
Marginal R^2	0.039			0.075		
Conditional R^2	0.132			0.316		
**Survey data**						
Confusability	6.06	1.92	6.26 E-04	7.55	3.46	4.67 E-02
# Sites	0.923	0.180	5.89 E-07	0.591	0.194	2.57 E-03
# Surveys	-0.168	0.016	9.07 E-21	-0.147	0.018	2.60 E-14
Marginal R^2	0.301			0.203		
Conditional R^2	NA (Fixed model)			0.352		

S2 Table 2. Results of mixed effects models for 50% phenometrics.

	Best model using survey data			Best model using incidental data		
Parameter	Estimate	SE	p value	Estimate	SE	p value
Adult diapause (0/1)	189.3	12	3.14 E-17	157.9	13.9	1.02 E-09
Pupal diapause (0/1)	226.6	11	1.50 E-20	186.3	12.2	3.89 E-14
Larval diapause (0/1)	233.4	9.9	1.58 E-23	194.6	10.9	4.84 E-14
Migrant (0/1)	261.2	11.7	6.56 E-22	215.9	16.4	1.47 E-11
Locally common (0/1)	-21.8	9.9	3.42 E-02	NA	NA	NA
Host breadth index	NA	NA	NA	10.7	5.4	6.22 E-02
Marginal R^2	0.4			0.37		
Conditional R^2	0.57			0.6		

S2 Figure 1: Species-specific boxplots of the difference between 10% DOY estimates from observation data and “day 0” estimates from regional field guides. The vertical line indicates an expected start of flight season based on a “Day 0” value estimated a week prior to the beginning of any flight period identified from sources (dark vertical line shows one week post-day 0). Incidental metrics in green, survey metrics in blue.

S2 Figure 2: Boxplots of estimated phenometrics by overwinter stage or strategy for each dataset. Survey phenometrics estimated from GAMs in blue and incidental phenometrics estimated using quantiles in green. Panel A shows emergence phenology (10% metrics) and panel B shows mid-season phenology (50% metrics).

S2 Figure 1

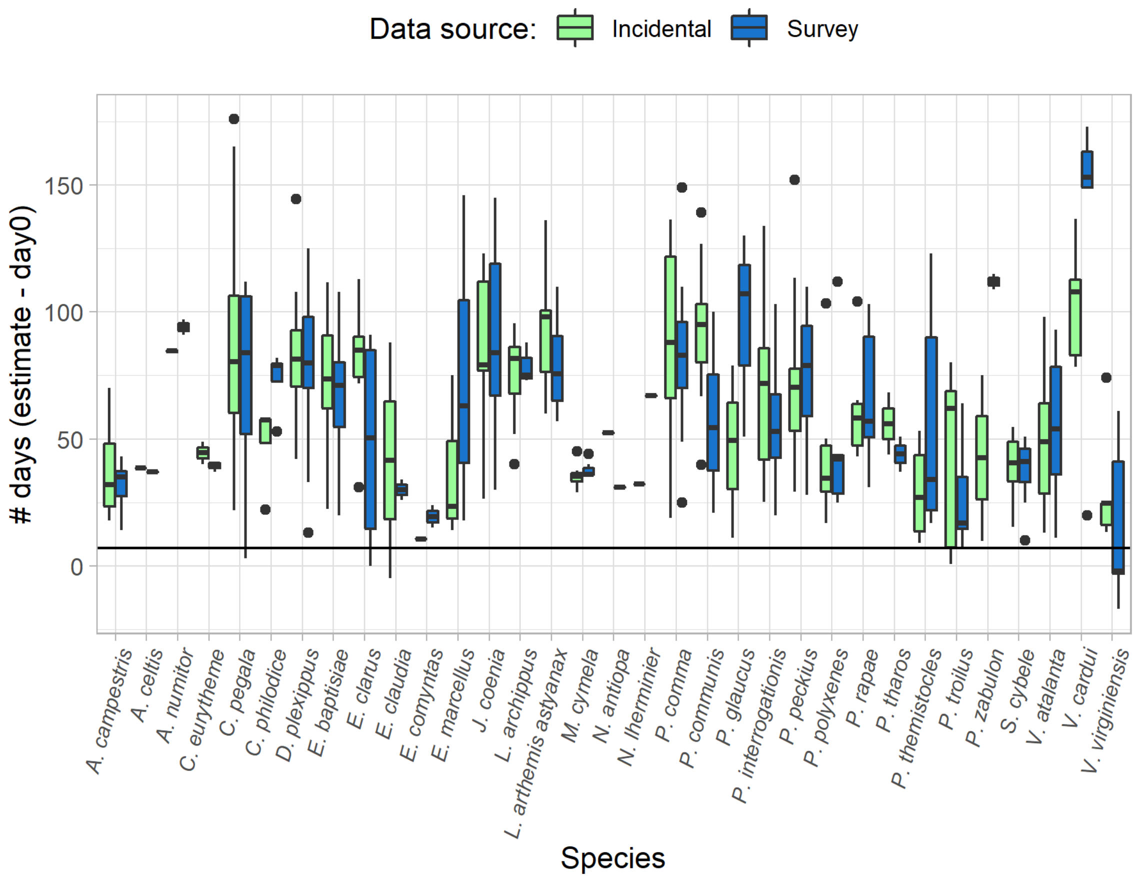

Data source:
Incidental
Survey
days (estimate - day0)
150
100
50
0
A. campestris
A. celtis
A. numitor
C. eurytheme
C. pegala
C. philodice
D. plexippus
E. baptisiae
E. clarus
E. claudia
E. comyntas
E. marcellus
J. coenia
L. archippus
L. arthemis astyanax
M. cymela
N. antiopa
N. lherminier
P. comma
P. communis
P. glaucus
P. interrogationis
P. peckius
P. polyxenes
P. rapae
P. tharos
P. themistocles
P. troilus
P. zabulon
S. cybele
V. atalanta
V. cardui
V. virginiensis
Species

S2 Figure 2

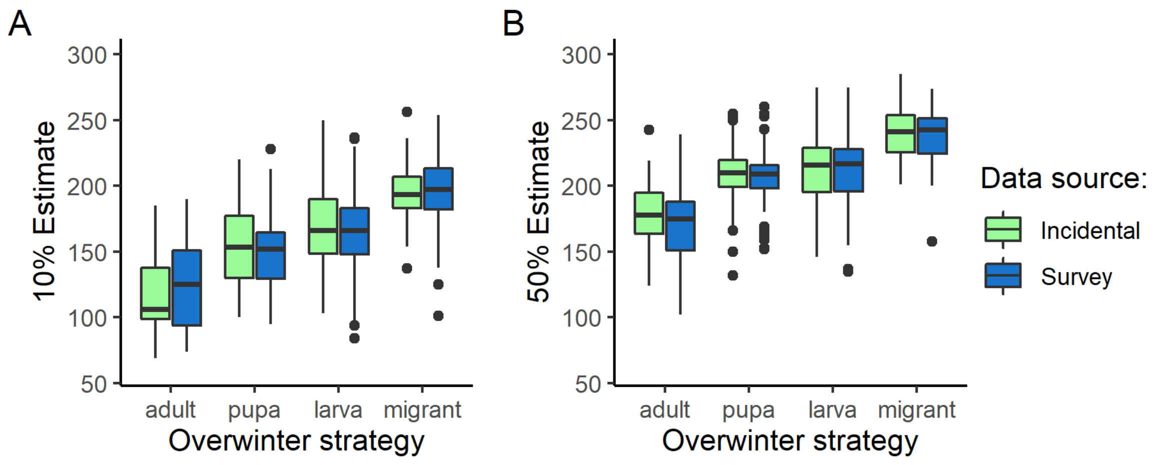
A
10% Estimate
300
250
200
150
100
50
adult
pupa
larva
migrant
Overwinter strategy
B
50% Estimate
300
250
200
150
100
50
adult
pupa
larva
migrant
Overwinter strategy
Data source:
Incidental
Survey
